# Five‐year outcomes of the STOP‐AD randomised controlled trial: Long‐term effects of early emollient therapy in high‐risk infants

**DOI:** 10.1111/pai.70517

**Published:** 2026-11-03

**Authors:** Molly L. Cremin, Sadhbh Hurley, Claire O'Halloran, Maria De los Angeles Gonzalez, Carol Ni Chaoimh, Roberto Velasco Zuniga, Juan Trujillo, Alan D. Irvine, Jonathan O'B Hourihane

**Affiliations:** ^1^ Irish Centre for Maternal and Child Health Research (INFANT) HRB Clinical Research Facility Cork (CRF‐C) Cork Ireland; ^2^ Paediatrics and Child Health University College Cork Cork Ireland; ^3^ Paediatrics Cork University Hospital Cork Ireland; ^4^ Clinical Medicine Trinity College Dublin Dublin Ireland; ^5^ Paediatrics and Child Health Royal College of Surgeons in Ireland Dublin Ireland; ^6^ Children's Health Ireland Dublin Ireland

**Keywords:** atopic dermatitis, emollient, prevention, randomised controlled trial, skin barrier

## Abstract

**Background:**

Early‐life skin barrier enhancement has been proposed as a strategy to prevent atopic dermatitis (AD), but results from emollient prevention trials have been inconsistent, and long‐term outcomes remain poorly defined. The STOP‐AD trial previously demonstrated a reduction in AD incidence following brief early emollient use in high‐risk infants. Whether these effects persist beyond infancy is unknown.

**Methods:**

This study reports the blinded five‐year follow‐up of the STOP‐AD single‐centre, randomised controlled trial. Term infants at high risk of AD were randomised within 4 days of birth to either twice‐daily whole‐body emollient application for the first 8 weeks of life or to standard routine skin care. At 5 years, participants underwent blinded, in‐person clinical assessment. AD was diagnosed using UK Working Party Diagnostic Criteria, and disease severity was assessed using SCORAD. Primary outcome was point prevalence of AD at 5 years. Secondary outcomes included AD severity. Complete‐case analyses were supplemented by multiple imputation to assess the impact of missing data.

**Results:**

A total of 177 children completed the five‐year follow‐up. The point prevalence of AD at 5 years was lower in the intervention group than in the control group (12.3% vs. 24.0%; RR 0.52, 95% CI 0.26–1.02; *p* = .048). Among children with AD, disease severity was also lower in the intervention group (median SCORAD 12.0 [IQR 8.2–15.2] vs. 19.6 [IQR 11.8–23.4]; *p* = .034). Multiple imputation to account for missing outcome data produced a similar treatment effect (OR 0.45, 95% CI 0.19–1.03; *p* = .060), although with greater uncertainty.

**Conclusions:**

Early, short‐term emollient use initiated in the neonatal period was associated with lower AD prevalence and severity at 5 years in high‐risk children. Although the effect estimate remained similar following multiple imputation, uncertainty due to attrition remained. These findings suggest that brief early‐life barrier support may influence longer‐term AD outcomes and support further investigation of the timing and formulation of preventive skin‐barrier interventions.

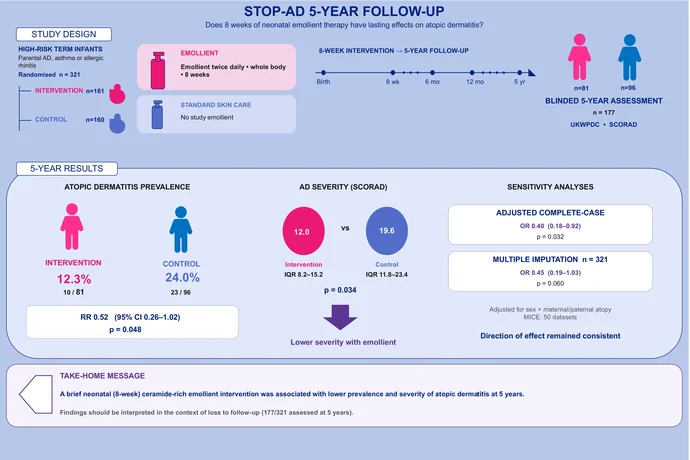

## Graphical abstract

In high‐risk infants, twice‐daily emollient use for the first 8 weeks of life was associated with lower atopic dermatitis prevalence and severity at 5 years. These findings suggest that brief neonatal skin‐barrier support may influence longer‐term atopic dermatitis outcomes.

**Key message**

Brief neonatal emollient therapy in high‐risk infants was associated with sustained reductions in both atopic dermatitis prevalence and severity at 5 years, suggesting that early skin‐barrier intervention may influence longer‐term AD outcomes.

## 1 INTRODUCTION

Atopic dermatitis (AD), or eczema, is one of the most prevalent chronic inflammatory skin conditions in childhood, affecting up to 20% of children worldwide.^1^, ^2^ The pathophysiology of AD is multifactorial, involving epidermal barrier dysfunction, immune dysregulation, dysbiosis and genetic susceptibility, most notably, loss‐of‐function mutations in the filaggrin (*FLG*) gene.^3^ Evidence suggests that a defective skin barrier may precede and contribute to the development of AD, supporting the hypothesis that early barrier enhancement could play a preventive role in atopic disease.^4^, ^5^ Identifying a simple, low‐cost preventative strategy could substantially reduce this impact.

The Short‐Term Topical Application to Prevent Atopic dermatitis (STOP‐AD) trial, a single centre randomised, blinded, controlled trial found that early application of a humectant and ceramide‐rich emollient from the first week of life to 8 weeks reduced the cumulative incidence of AD by 50% at 6 months and 29% at 12 months, compared with untreated controls.^6^

However, other large multicentre trials have not replicated these findings, possibly due to differences in study design, emollient formulation, and timing or frequency of application. Importantly, almost all existing studies have focused on outcomes within the first 12–24 months of life; only two trials have reported follow‐up beyond 24 months, and of these, just one included in‐person clinical assessment. There is a critical gap in understanding the long‐term durability of any preventative or protective effects of early emollient use.^7^, ^8^, ^9^, ^10^

We report on the five‐year follow‐up of participants from the STOP‐AD trial. We sought to determine whether early, short‐term prophylactic emollient application during early infancy affects the prevalence and severity of AD at 5 years of age.

## 2 METHODOLOGY

### 2.1 Original study design and participants

This study represents a long‐term follow‐up cohort derived from the original STOP‐AD randomised controlled trial, a single‐centre, two‐arm, randomised controlled trial conducted at Cork University Maternity Hospital (CUMH) and Cork University Hospital (CUH), Ireland. The study was approved by the Clinical Research Ethics Committee of the Cork Teaching Hospitals (Ref: ECM 5 (2) 18/12/18), and the trial's primary outcomes were previously published in 2023.^6^

Initial recruitment took place at CUMH between April 2019 and November 2020 and included term newborn infants at high risk of AD, defined by having at least one parent with a self‐reported history of AD, asthma, or allergic rhinitis.

Infants were randomised in a 1:1 allocation ratio within the first 4 days of life to receive either twice‐daily application of emollient for the first 8 weeks of life in the intervention group (IG) or standard skin care advice in the control group (CG). Randomisation was performed using block randomisation with varying block sizes of 2, 4, and 6 and implemented using sequentially numbered, opaque, sealed envelopes. Allocation was concealed by the study project manager, who had no patient contact or access to participant data.

Outcome assessment at the 5‐year follow‐up was performed by investigators who were blinded to treatment allocation. The IG received instructions to apply AVEENO® Dermexa Fast & Long‐Lasting Balm (Johnson & Johnson Santé Beauté France) twice daily to the entire body for the first 8 weeks of life. The CG received standard routine skin care advice. No further intervention, specific skin care guidance, or restrictions on emollient use were provided beyond this period prior to the 5‐year follow‐up. The target sample size of 242 infants (121 per group) provided 80% power at a 95% confidence level to detect a 50% reduction in cumulative AD at 12 months (from 30% to 15%). To allow for anticipated withdrawal and loss to follow‐up, recruitment continued until 321 infants had been randomised.

For the 5‐year follow‐up the study was approved by the Clinical Research Ethics Committee of the Cork Teaching Hospitals (ECM 5 (2) 18/12/18 and ECM 3 05/03/2024). All participants from the original cohort who had consented to being recontacted were approached. Participating carers completed an online questionnaire using the CASTOR electronic data capture system and if they agreed, a clinical review was conducted to confirm reported outcomes.

### 2.2 Blinding

Research personnel conducting follow‐up assessments at 5 years were blinded to original group allocation. Parents were instructed, on booking, not to reveal their infant's assigned group during telephone or in‐person contact with the blinded clinician.

### 2.3 Data collection and clinical assessments

#### 2.3.1 Online survey

Data collected comprised demographic data, personal and family medical history, and information on symptoms of AD.

#### 2.3.2 Clinical review

This included anthropometric measurements and a standardised skin examination for signs of AD. Trans epidermal water loss (TEWL) was measured on the volar forearm using a closed‐chamber vapometer (Delfin Technologies, UK) after a minimum 10‐min acclimatisation period in a controlled clinical environment with standardised temperature and humidity conditions, and measurements were taken in accordance with manufacturer guidance.

#### 2.3.3 Atopic dermatitis assessment

Survey responses were reviewed during the clinical visit, and parents were asked about any history or current symptoms of AD. Participants were assessed by trained blinded study assessors using the UK Working Party Diagnostic Criteria (UKWPDC); where there was uncertainty regarding diagnosis or disease severity, a second investigator reviewed the participant and a consensus assessment was reached. Disease severity was assessed using the SCORing Atopic Dermatitis (SCORAD) index^11^, ^12^ SCORAD severity scoring was applied only to participants with confirmed atopic dermatitis, as defined by the UKWPDC.

### 2.4 Study outcomes

The primary outcome was the point prevalence of atopic dermatitis at 5 years of age. Secondary outcomes included severity of AD (measured by SCORAD).

### 2.5 Data analysis

#### 2.5.1 Primary analysis

Analyses were conducted according to original randomisation group. The primary analysis included participants with available 5‐year outcome data (complete‐case population; *n* = 177). Baseline characteristics were summarised using descriptive statistics. Continuous variables are presented as mean (SD) and were compared between treatment groups using Welch's two‐sample *t*‐test. Categorical variables are presented as *n* (%) and were compared using Fisher's exact test.

The primary outcome, AD point prevalence at 5 years, was compared between intervention and control groups using Pearson's chi‐square test. Relative risks (RRs) with 95% confidence intervals (CIs) were calculated. Statistical significance was defined as a two‐sided *p* < .05.

#### 2.5.2 Sensitivity analyses

To assess the potential impact of attrition and missing outcome data, several sensitivity analyses were performed. Baseline characteristics of participants who completed the 5‐year follow‐up were compared with those of the original randomised cohort (*n* = 321). Logistic regression was used to assess whether AD status at 6 or 12 months was associated with attendance at the 5‐year follow‐up. These associations were examined separately within each treatment group, with interaction terms used to assess whether associations differed by treatment group. A multivariable complete‐case logistic regression model assessed the association between treatment allocation and AD at 5 years, adjusting for sex, maternal atopy, and paternal atopy.

A further sensitivity analysis assessed 5‐year AD accounting for adherence and contamination during the 8 week intervention period, including participants in the intervention group reporting emollient use ≥4 days/week and those in the control group reporting use ≤3 days/week.

Multiple imputation by chained equations (MICE) was subsequently performed in the original randomised cohort (*n* = 321), generating 50 imputed datasets. The imputation model included 5‐year AD status, treatment allocation, sex, maternal and paternal atopy, FLG status, and AD status at 6 and 12 months. Estimates across imputed datasets were pooled using Rubin's rules.

#### 2.5.3 Exploratory analysis

Exploratory analysis included assessment of longitudinal AD phenotypes using AD status at 6 months, 12 months and 5 years. Participants were categorized according to patterns of AD onset and persistence over follow‐up, and phenotype distributions were compared descriptively between treatment groups.

## 3 RESULTS

### 3.1 Participant flow and attrition

A total of 321 participants were originally randomised in the STOP‐AD trial, of whom 257 had outcome data available at 12 months and were eligible for the 5‐year follow‐up. Of these, 243 (95%) consented to participate in continued follow‐up. At 5 years, 179 participants attended for assessment; two were excluded from the primary analysis as the clinician became unblinded during clinical review. The final analysis cohort therefore comprised 177 participants (81 intervention and 96 control) (Figure 1).

**FIGURE 1:**
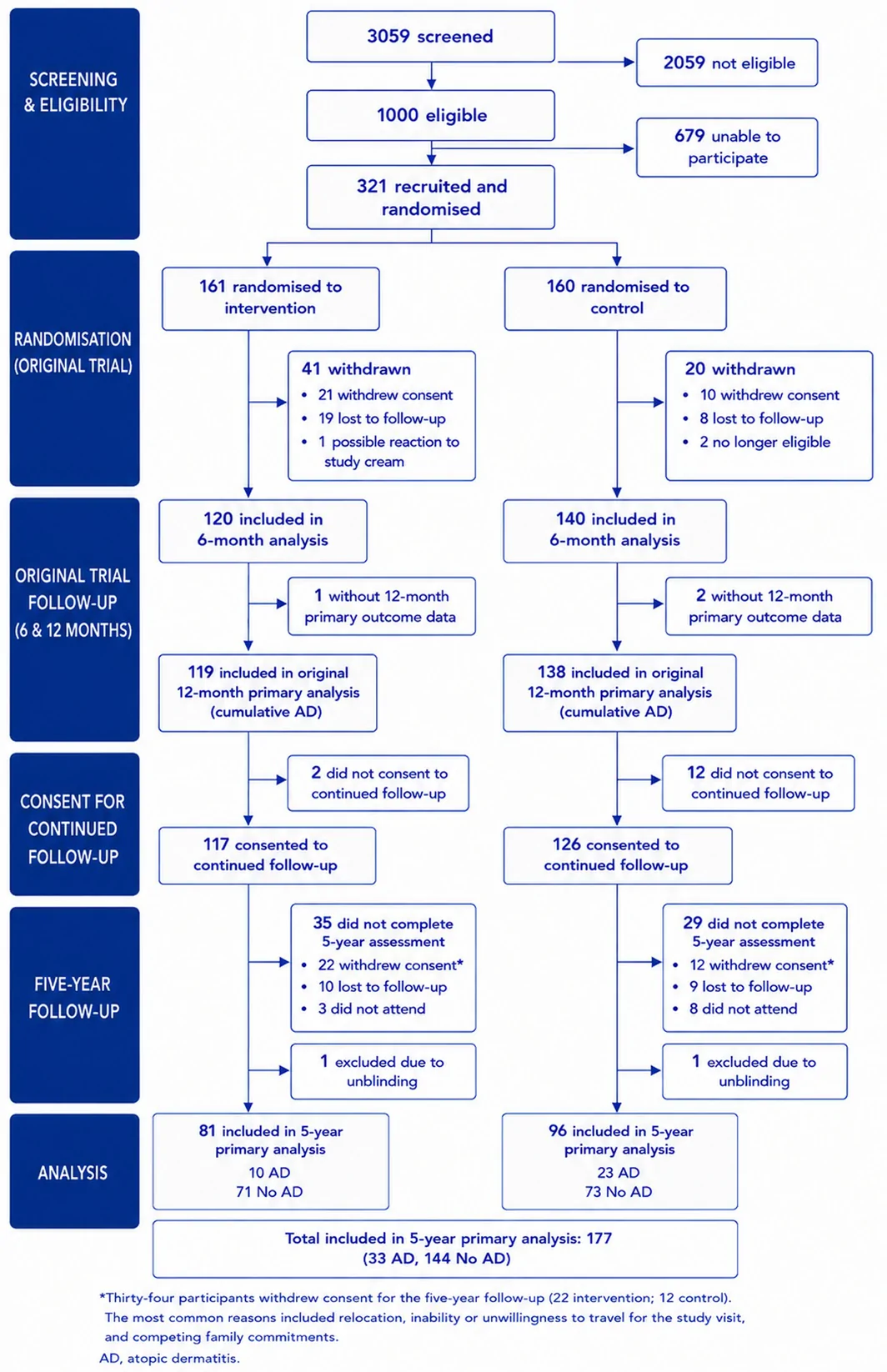
Participant flow through the original STOP‐AD trial and five‐year follow‐up. Participant flow from screening and randomisation through the original 6‐ and 12‐month assessments and subsequent 5‐year follow‐up. Of 321 participants originally randomised, 177 were included in the five‐year primary analysis (81 intervention; 96 control).

Baseline characteristics of the 5‐year cohort were generally similar between treatment groups (Table 1). Baseline characteristics were also compared between participants who completed and did not complete 5‐year follow‐up within each treatment group (Table S1). Logistic regression demonstrated that neither AD at 6 months nor AD at 12 months was associated with attendance at the 5‐year assessment (Table S2), providing no evidence that attrition was associated with earlier AD status.

**TABLE 1:** Characteristics of participants assessed at 5 years.

Characteristic	Intervention (*n* = 81)^a^	Control (*n* = 96)^a^	*p*‐value^b^
Infant characteristics			
Male sex	41 (50.6%)	47 (49.0%)	0.881
Gestational age, weeks	39.8 (1.1)	39.5 (1.1)	0.033
Birth weight, kg	3.62 (0.40)	3.56 (0.46)	0.350
Parity	1.7 (0.8)	1.7 (0.8)	0.763
Caesarean section	28 (34.6%)	32 (33.3%)	0.875
Infant feeding			0.905
Breast milk only	28 (43.8%)	33 (41.3%)	
Formula only	23 (35.9%)	32 (40.0%)	
Combined feeding	13 (20.3%)	15 (18.8%)	
Parental characteristics			
Maternal age, years	33.5 (4.0)	34.4 (4.1)	0.2
Paternal age, years	36.0 (5.8)	36.1 (4.7)	>0.9
Maternal history of atopy	57/81 (70%)	61/96 (64%)	0.04
Paternal history of atopy	51/81 (63%)	71/96 (74%)	0.14
Maternal White ethnic background	78/81 (96%)	86/96 (90%)	0.15
Paternal White ethnic background	74/81 (91%)	89/96 (93%)	0.8
Environmental exposures			
Living rurally	35 (43.2%)	47 (49.0%)	0.454
Pets in the home	31 (38.3%)	34 (35.4%)	0.755
Mold in the home	34 (42.0%)	25 (26.0%)	0.037

^a^ Data are presented as *n*/*N* (%) for categorical variables and mean (SD) for continuous variables.

^b^ Fisher's exact test; Welch Two Sample *t*‐test.

### 3.2 Primary outcome

#### 3.2.1 AD at 5 years

At 5 years, AD was diagnosed in 10 of 81 children (12.3%) in the intervention group compared with 23 of 96 (24.0%) in the control group (RR 0.52, 95% CI 0.26–1.02; *p* = .048) (Figure 2).

**FIGURE 2:**
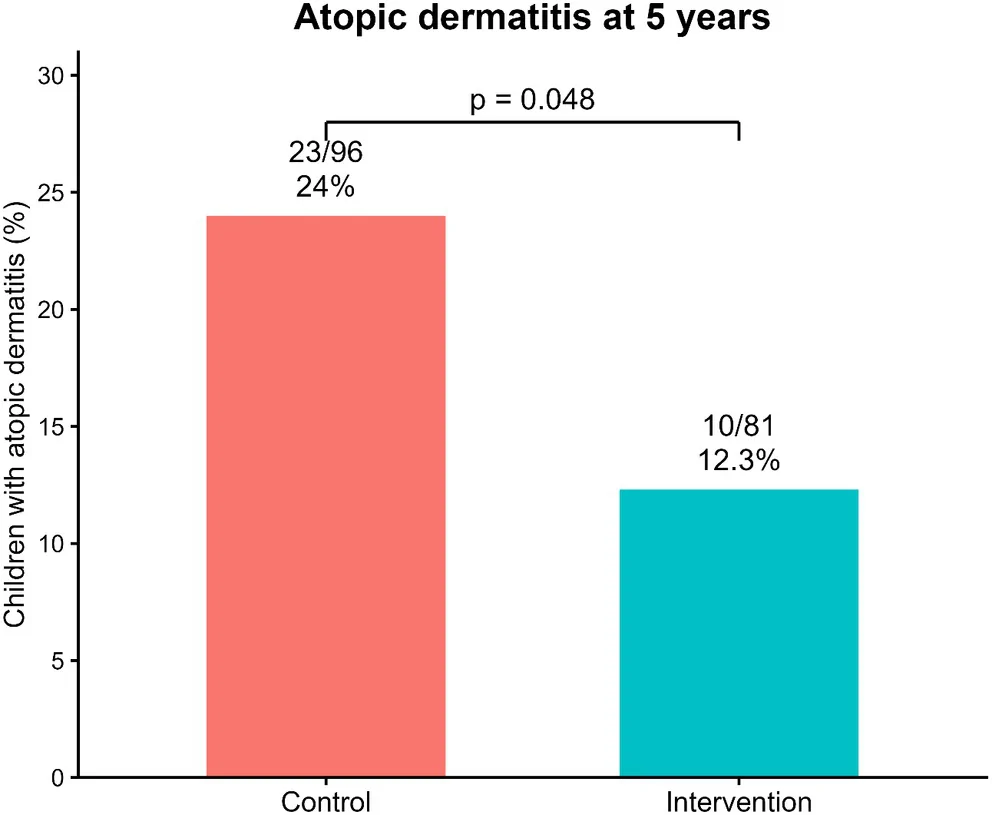
Point prevalence of atopic dermatitis (AD) at 5 years among participants who completed the 5‐year assessment (*n* = 177). AD was present in 23/96 (24.0%) children in the control group and 10/81 (12.3%) in the intervention group. The *p*‐value represents the between‐group comparison.

### 3.3 Secondary outcome

#### 3.3.1 AD severity

Among children with AD at 5 years (intervention, *n* = 10; control, *n* = 23), disease severity was lower in the intervention group. Median SCORAD was 12.0 (IQR 8.2–15.2) in the intervention group compared with 19.6 (IQR 11.8–23.4) in the control group (Wilcoxon rank‐sum test, *W* = 169.5; *p* = .034) (Figure 3).

**FIGURE 3:**
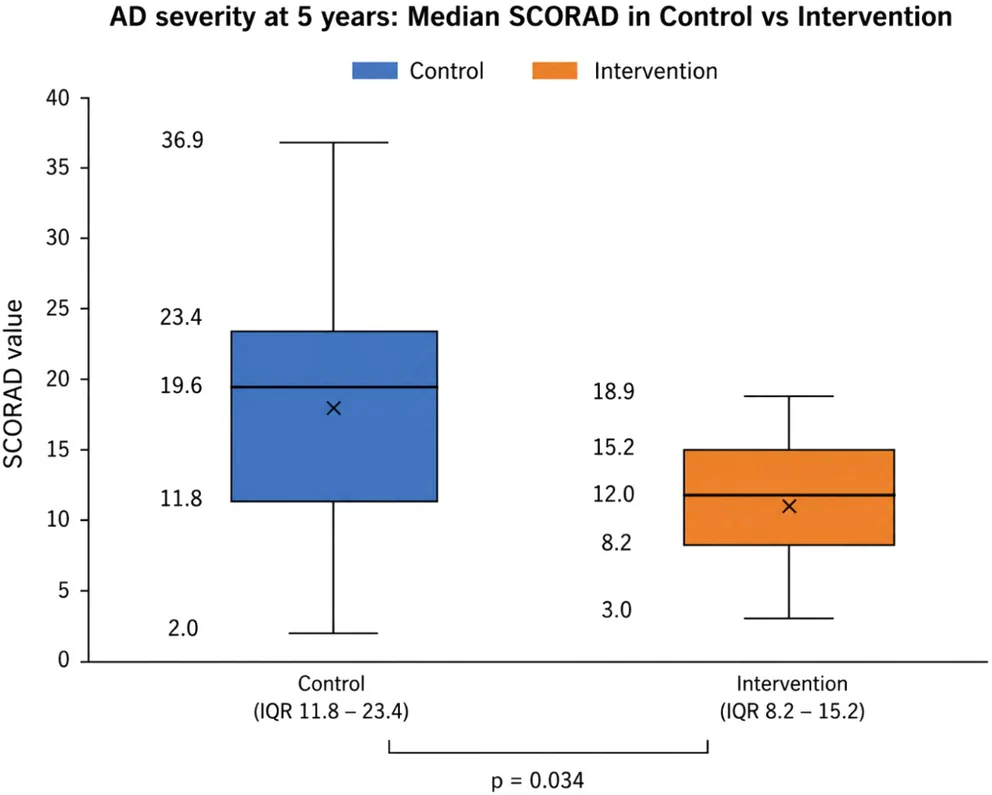
Boxplot shows the distribution of SCORAD values at 5 years in the IG vs. CG. SCORAD scores among children with AD at the five‐year assessment. Boxes represent the interquartile range (IQR), horizontal lines represent the median, and whiskers represent the observed range. AD severity was lower in the intervention group than in the control group (*p* = .034; Wilcoxon rank‐sum test).

### 3.4 Sensitivity and exploratory analysis

#### 3.4.1 Sensitivity analysis

Multivariable complete case analysis was completed to explore the association between treatment allocation and AD at 5 years after adjustment for sex, maternal atopy, and paternal atopy (aOR 0.40, 95% CI 0.18–0.92; *p* = .032).

In further sensitivity analysis accounting for adherence and contamination during the 8‐week intervention period, AD at 5 years was present in 6/47 (12.8%) of those in the intervention group who reported emollient use ≥4 days/week compared with 15/44 (34.1%) in the control group who reported emollient use on ≤3 days/week (RR 0.37, 95% CI 0.16–0.88; *p* = .024).

To assess the potential impact of missing outcome data, multiple imputation was performed using the fully randomised cohort (*n* = 321). Following multiple imputation, the estimated treatment effect remained similar in magnitude and direction although with reduced precision (OR 0.45, 95% CI 0.19–1.03; *p* = .060) (Figure 4).

**FIGURE 4:**
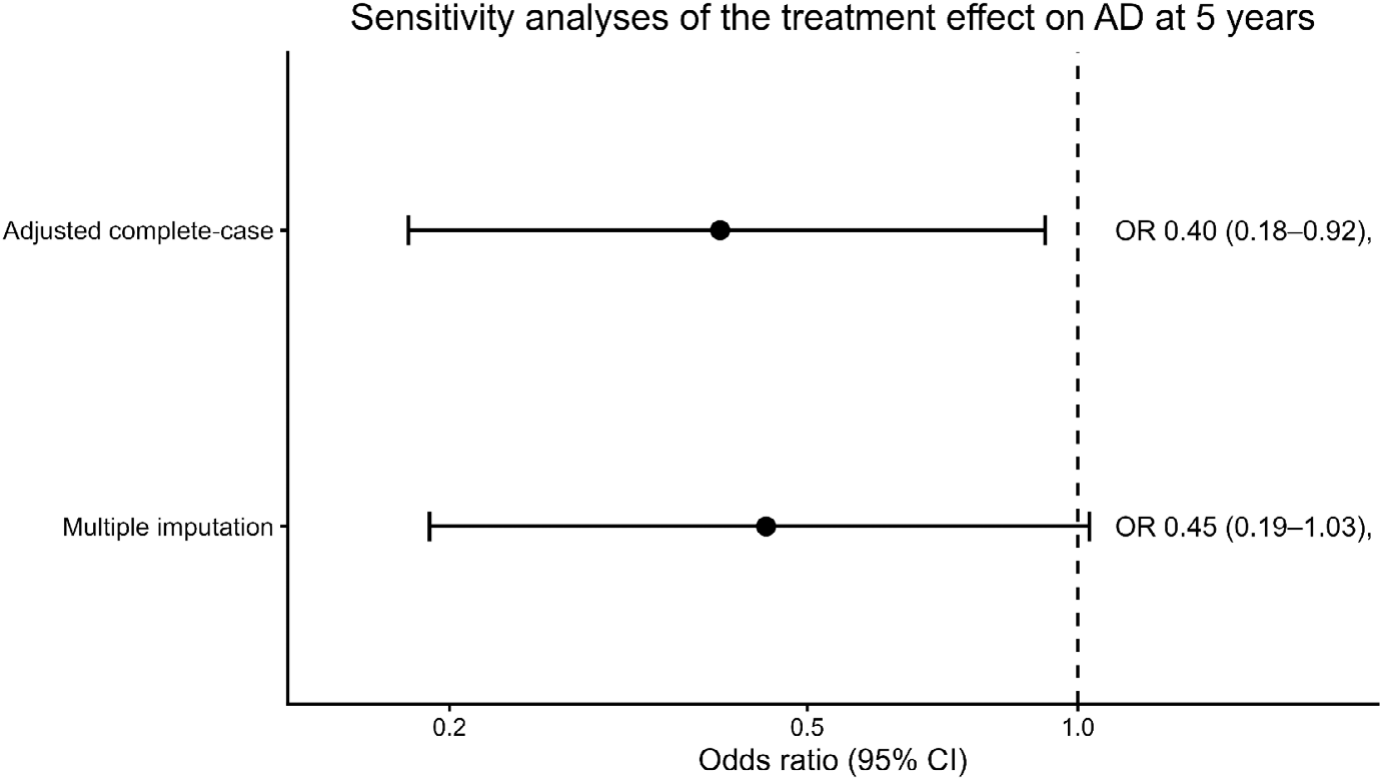
Forest plot showing odds ratios (ORs) and 95% confidence intervals (CIs) for the association between treatment allocation and AD at 5 years across adjusted complete‐case and multiple imputation analyses. The adjusted model included sex, maternal atopy, and paternal atopy. The dashed vertical line represents the null value (OR = 1).

#### 3.4.2 Further exploratory analysis

Exploratory analysis of AD trajectories at 6 months, 12 months, and 5 years demonstrated numerically lower proportions of early‐onset persistent, early‐onset resolving, and late‐onset AD in the intervention group, with a greater proportion remaining free of AD at all assessments (Table S3).

## 4 DISCUSSION

To our knowledge, this study represents the first in‐person, blinded, five‐year follow‐up of a randomised controlled trial evaluating early, short‐term emollient use in high‐risk infants for the prevention of AD. Consistent with the original STOP‐AD trial, we observed a lower point prevalence of AD at 5 years among children who had received the early emollient intervention compared with controls. This association was statistically significant in the primary complete‐case analysis (*p* = .048).

Given the substantial attrition, additional analyses were undertaken to assess the robustness of this finding. Adjustment for sex and parental atopy did not alter the association between treatment allocation and AD at 5 years (*p* = .032). A sensitivity analysis accounting for adherence and contamination during the 8‐week intervention period demonstrated a similar direction of effect, with a lower prevalence of AD at 5 years among participants meeting the predefined adherence criteria. Multiple imputation to account for missing outcome data showed a similar protective effect, although with reduced precision and this analysis did not reach statistical significance (*p* = .06). Overall, these findings suggest that the effect of early emollient use may persist to 5 years; however, the substantial loss to follow‐up introduces uncertainty and the results should be interpreted with caution.

Among children with AD at 5 years, disease severity, measured by SCORAD, was also lower in the intervention group. Interestingly, no difference in AD severity was seen between groups at 12 months in the original STOP‐AD study. AD is increasingly recognized as a heterogeneous disease with distinct longitudinal patterns of onset, persistence and resolution throughout childhood.^13^ In this context, the difference in severity observed at 5 years may reflect differences in the pattern of AD over time rather than simply disease severity at a single time point.

Our exploratory longitudinal analysis supports this possibility. The difference in AD at 5 years appeared to be mainly among children with persistent disease, while late‐onset AD occurred at a similar frequency in the two groups. This raises the possibility that early barrier intervention may have had a greater effect on the development or persistence of early‐onset AD, rather than preventing AD arising for the first time later in childhood. This may also help explain why a difference in disease severity was apparent at 5 years but not at 12 months. However, the numbers contributing to both the AD severity and longitudinal phenotype analyses were small, and these findings should therefore be considered exploratory.

### 4.1 Comparison with previous studies

These findings differ from those of larger‐scale trials, which did not demonstrate a preventive effect of early emollient use on AD. Differences in study populations, intervention characteristics, and outcome ascertainment may therefore have contributed to the variation in long‐term outcomes observed across trials.

The BEEP trial, which followed participants to 5 years of age, reported no significant reduction in AD or other atopic outcomes at any assessed time point (12 months, 24 months, or 5 years). However, the five‐year outcomes in BEEP were based on parental reports rather than blinded clinical assessment, which may have limited diagnostic precision and the accurate characterization of disease persistence or severity.^14^

Similarly, the PreventADALL study, which followed participants to 36 months of age, did not demonstrate a protective effect of emollient use at any time point. In contrast to the BEEP trial, clinical assessments were performed at later follow‐up visits, but standardised, blinded evaluations of AD severity were not reported, limiting direct comparison with the present findings.^10^

Few previous trials have extended follow‐up beyond 2 years, and even fewer have incorporated blinded, in‐person assessment at later time points. The use of blinded, standardised clinical assessment at 5 years in STOP‐AD therefore represents a notable methodological characteristic of STOP‐AD and allows assessment of long‐term outcomes using standardised clinical criteria.

### 4.2 Relation to emerging evidence

In 2025, the CASCADE trial reported a significant reduction in AD incidence at 24 months following early emollient use initiated before 9 weeks of age in the general infant population (RR = 0.84; 95% CI 0.73–0.97; *p* = .02). Notably, CASCADE was not limited to a high‐risk cohort, suggesting that the potential benefit of early barrier intervention may extend beyond infants at high familial risk of AD.^15^

Despite differences in study populations, both CASCADE and STOP‐AD share key design features, including early initiation of emollient therapy and the use of emollients designed to support epidermal barrier function. However, the CASCADE intervention was pragmatic, and although it incorporated a ceramide‐containing emollient, it was not restricted to a single formulation, and switching was observed over time. This may be important, as recent evidence suggests that emollients should not be considered functionally equivalent, with differences in lipid composition and formulation potentially influencing their effects on barrier repair.^16^

In both studies, early emollient interventions were associated with reductions in AD beyond the immediate intervention period, despite differences in population risk and duration of follow‐up. The timing of intervention may be particularly important, as barrier dysfunction and immune activation may begin very early in life, potentially narrowing the window for effective primary prevention.^16^ Consistent with this, Kim et al. proposed that preventive interventions may be most effective during the early postnatal period while the skin barrier is still maturing.^17^ This may be particularly relevant to STOP‐AD, where treatment was initiated within the first days of life.

Emerging mechanistic data further suggest that the effects of emollient therapy may extend beyond hydration. Studies comparing trilipid formulations with paraffin‐based emollients have demonstrated improved barrier function, measured by reductions in TEWL, as well as potential modulation of immune responses, including increased IgG4 and reduced IgE levels. These findings support the concept that barrier‐enhancing formulations may influence skin integrity and downstream sensitisation pathways.^18^, ^19^

The PACI trial and its three‐year follow‐up, PACI‐ON, demonstrated that early enhanced treatment of AD reduced subsequent food allergy, with effects persisting beyond the intervention period. Although mechanistically different from STOP‐AD, these findings further support the potential importance of early‐life skin‐directed intervention in modifying later atopic outcomes.^20^, ^21^

Taken together, these observations suggest that both the timing and formulation of early barrier interventions may be important determinants of their effectiveness. The findings from CASCADE, together with the present five‐year STOP‐AD results, support further investigation of whether targeted barrier support initiated early in life can modify longer‐term AD outcomes.

## 5 STRENGTHS AND LIMITATIONS

Strengths of this study include the long duration of follow‐up and blinded, in‐person clinical assessment at 5 years, enabling AD diagnosis and severity assessment using standardised criteria.

The principal limitation is the substantial loss to follow‐up, with 177 of the original 321 randomised participants included in the primary analysis and lower retention in the intervention group. This introduces the possibility of differential attrition and selection bias, particularly as families were aware of their original treatment allocation. Although baseline comparisons and analyses of predictors of attendance were undertaken, and multiple imputation produced an effect estimate in the same protective direction, the imputed analysis did not reach statistical significance. The long‐term treatment effect should therefore be interpreted cautiously.

The single‐centre, high‐risk population may also limit generalisability. Emollient use beyond 12 months and topical corticosteroid use were not systematically recorded, and the small number of children with active AD limited the severity analysis. Finally, the five‐year and longitudinal phenotype analyses were not prespecified in the original trial protocol and should be considered exploratory.

## 6 CONCLUSIONS

The original STOP‐AD study demonstrated that early initiation of short‐term emollient use in high‐risk infants reduced the point prevalence of AD at 6 and 12 months, and this report suggests that this benefit may persist to 5 years, with the prevalence and severity of AD at 5 years being lower in the intervention group compared with controls. STOP‐AD helps contextualize the mixed findings of previous prevention trials by suggesting that early, short‐term barrier enhancement in high‐risk infants may influence longer‐term AD outcomes. Future research should focus on optimizing AD prevention strategies, with particular attention to emollient formulation, timing and adherence, and risk stratification to maximize preventive benefits.

## AUTHOR CONTRIBUTIONS

**Molly L. Cremin:** Writing – original draft; methodology; investigation. **Sadhbh Hurley:** Writing – review and editing; supervision. **Claire O'Halloran:** Resources; investigation; project administration. **Maria De los Angeles Gonzalez:** Investigation. **Carol Ni Chaoimh:** Conceptualization; investigation; methodology. **Roberto Velasco Zuniga:** Formal analysis; data curation. **Juan Trujillo:** Conceptualization; investigation; writing – review and editing; supervision. **Alan D. Irvine:** Conceptualization; investigation; funding acquisition; writing – review and editing; supervision. **Jonathan O'B Hourihane:** Conceptualization; investigation; funding acquisition; writing – review and editing; visualization; supervision.

## FUNDING INFORMATION

City of Dublin Skin and Cancer Hospital Charity.

## CONFLICT OF INTEREST STATEMENT

The emollient used in this study was provided by its manufacturer Johnson and Johnson Santé Beauté France (JJSBF) as benefit‐in‐kind. JJSBF did not have any role in the trial design. JJSBF provided funding for a sub‐study within this study, which includes the analysis of the microbiome and inflammatory biomarkers in a subgroup of the study. This is separate from the study's primary funding source.

## Supporting information


**Table S1:** Baseline characteristics of participants who completed the 5‐year follow‐up compared with those lost to follow‐up, stratified by Randomisation group.
**Table S2:** Association between atopic dermatitis status at 6 and 12 months and participation at the 5‐year follow‐up.
**Table S3:** Longitudinal atopic dermatitis phenotypes by treatment allocation.

**Data S1:** CONSORT 2025 checklist of information to include when reporting a randomised trial*.

## Data Availability

The data that support the findings of this study are available on request from the corresponding author. The data are not publicly available due to privacy or ethical restrictions.
