# Supplementary material for: Five‐year outcomes of the STOP‐AD randomised controlled trial: Long‐term effects of early emollient therapy in high‐risk infants
Source: Pediatr Allergy Immunol. 2026 Nov 3;37(11):e70517. doi: 10.1111/pai.70517 (PMC13728885; doi:10.1111/pai.70517)
Supplement: Supplementary file 1 [file PAI-37-e70517-s001.docx]

**Supplementary Table 1.**

Baseline characteristics of participants who completed the 5-year follow-up compared with those lost to follow-up, stratified by randomisation group

| **Characteristic** | **Control Follow-up (n = 96)** | **Control Lost to follow-up (n = 64)** | **P-value** | **Intervention Follow-up (n = 81)** | **Intervention Lost to follow-up (n = 80)** | **P-value2** |
| --- | --- | --- | --- | --- | --- | --- |
| **Parental characteristics** |  |  |  |  |  |  |
| Maternal age, years | 34.86 (4.09) | 34.09 (5.69) | 0.35 | 33.99 (3.99) | 33.52 (4.79) | 0.505 |
| Paternal age, years | 36.54 (4.71) | 36.23 (6.51) | 0.746 | 36.53 (5.80) | 34.84 (5.44) | 0.06 |
| Maternal atopy | 60/96 (62.5%) | 48/64 (75.0%) | 0.12 | 57/81 (70.4%) | 54/80 (67.5%) | 0.735 |
| Paternal atopy | 71/96 (74.0%) | 37/63 (58.7%) | 0.056 | 51/80 (63.8%) | 51/80 (63.8%) | 1 |
| Maternal White ethnic background | 86/96 (89.6%) | 54/64 (84.4%) | 0.24 | 78/81 (96.3%) | 70/80 (87.5%) | 0.047 |
| Paternal White ethnic background | 89/96 (92.7%) | 54/64 (84.4%) | 0.12 | 73/81 (90.1%) | 71/80 (88.8%) | 0.609 |
| **Infant characteristics** |  |  |  |  |  |  |
| Female sex | 47/96 (49.0%) | 29/64 (45.3%) | 0.747 | 41/81 (50.6%) | 40/80 (50.0%) | 1 |
| Gestational age, weeks | 39.46 (1.13) | 39.62 (1.13) | 0.38 | 39.82 (1.14) | 39.62 (1.08) | 0.26 |
| Birth weight, kg | 3.56 (0.46) | 3.62 (0.44) | 0.34 | 3.62 (0.40) | 3.56 (0.45) | 0.373 |
| Caesarean delivery | 32/96 (33.3%) | 24/64 (37.5%) | 0.61 | 28/81 (34.6%) | 35/80 (43.8%) | 0.261 |
| Any breastfeeding at 8 weeks | 48/80 (60.0%) | 29/51 (56.9%) | 0.856 | 41/64 (64.1%) | 36/68 (52.9%) | 0.219 |
| **Environmental characteristics** |  |  |  |  |  |  |
| Rural residence | 47/96 (49.0%) | 35/64 (54.7%) | 0.515 | 35/81 (43.2%) | 29/80 (36.2%) | 0.422 |
| Pets in household | 34/96 (35.4%) | 33/64 (51.6%) | 0.05 | 31/81 (38.3%) | 39/80 (48.8%) | 0.205 |

*Data are presented as n/N (%) for categorical variables and mean (SD) for continuous variables. P-values were calculated using Fisher’s exact test for categorical variables and Welch’s two-sample t-test for continuous variables.*

**Supplementary Table 2. Association between atopic dermatitis status at 6 and 12 months and participation at the 5-year follow-up.**

*Data are presented as n/N (%). Odds ratios represent the odds of participation at the 5-year follow-up in participants with AD compared with those without AD at the corresponding timepoint, calculated separately for the intervention and control groups. OR, odds ratio; CI, confidence interval; AD, atopic dermatitis.*

**Supplementary Table 3. Longitudinal atopic dermatitis phenotypes by treatment allocation.**

*Early-onset persistent AD was defined as AD at 6 and/or 12 months with AD present at 5 years; early-onset resolving AD as AD at 6 and/or 12 months with no AD at 5 years; late-onset AD as no AD at 6 or 12 months with AD present at 5 years; and never AD as no AD at 6, 12 months, or 5 years. Data are presented as n (% within treatment group).*
