# Supplementary material for: Five‐year outcomes of the STOP‐AD randomised controlled trial: Long‐term effects of early emollient therapy in high‐risk infants
Source: Pediatr Allergy Immunol. 2026 Nov 3;37(11):e70517. doi: 10.1111/pai.70517 (PMC13728885; doi:10.1111/pai.70517)
Supplement: Supplementary file 2 [file PAI-37-e70517-s002.docx]

**CONSORT 2025 checklist of information to include when reporting a randomised trial***

| **Section / Topic** | **No** | **CONSORT 2025 checklist item description** | **Reported on page no.** |
| --- | --- | --- | --- |
| **Title and abstract** | | |  |
| Title and structured abstract | 1a | Identification as a randomised trial | 1 |
|  | 1b | Structured summary of the trial design, methods, results, and conclusions | 1-2 |
| **Open science** | | |  |
| Trial registration | 2 | Name of trial registry, identifying number (with URL) and date of registration | ClinicalTrials.gov, NCT03871998; registered 12 March 2019. |
| Protocol and statistical analysis plan | 3 | Where the trial protocol and statistical analysis plan can be accessed | The study protocol and statistical analysis plan are securely archived on a password-protected institutional server at University College Cork and are available from the corresponding author on reasonable request. |
| Data sharing | 4 | Where and how the individual de-identified participant data (including data dictionary), statistical code and any other materials can be accessed | De-identified participant data, the data dictionary are securely stored on password-protected institutional devices and may be made available from the corresponding author on reasonable request, subject to appropriate ethical and institutional approvals. |
| Funding and conflicts of interest | 5a | Sources of funding and other support (e.g., supply of drugs), and role of funders in the design, conduct, analysis and reporting of the trial | Primary funding: The City of Dublin Skin and Cancer Hospital Charity  Additional funding: Skin Research Institute of Singapore (A*STAR-BMRC-EDBH17/01/a0/004) The emollient used in this study was provided by its manufacturer Johnson and Johnson Santé Beauté France (JJSBF) as benefit- in-kind. JJSBF did not have any role in the trial design |
|  | 5b | Financial and other conflicts of interest of the manuscript authors | Conflicts of Interest statement |
| **Introduction** | | |  |
| Background and rationale | 6 | Scientific background and rationale | Introduction, pp. 2–3 |
| Objectives | 7 | Specific objectives related to benefits and harms | Introduction, p. 3 — objective was to determine whether early short-term prophylactic emollient application affected AD prevalence and severity at 5 years |
| **Methods** | | |  |
| Patient and public involvement | 8 | Details of patient or public involvement in the design, conduct and reporting of the trial | Not reported / no specific patient or public involvement reported |
| Trial design | 9 | Description of trial design including type of trial (e.g., parallel group, crossover), allocation ratio, and framework (e.g., superiority, equivalence, non-inferiority, exploratory) | Methods, p. 3 — long-term follow-up of a single-centre, two-arm RCT; 1:1 allocation. |
| Changes to trial protocol | 10 | Important changes to the trial after it commenced including any outcomes or analyses that were not prespecified, with reason | Methods/Limitations five-year and longitudinal phenotype analyses were not prespecified in the original trial protocol and are considered exploratory. |
| Trial setting | 11 | Settings (e.g., community, hospital) and locations (e.g., countries, sites) where the trial was conducted | Cork University Maternity Hospital/Cork University Hospital, Ireland. |
| Eligibility criteria | 12a | Eligibility criteria for participants | Methods, p. 3 term newborns at high risk of AD, defined by ≥1 parent with self-reported AD, asthma or allergic rhinitis. |
|  | 12b | If applicable, eligibility criteria for sites and for individuals delivering the interventions (e.g., surgeons, physiotherapists) | N/A |
| Intervention and comparator | 13 | Intervention and comparator with sufficient details to allow replication. If relevant, where additional materials describing the intervention and comparator (e.g., intervention manual) can be accessed | Methods, pp. 3–4 — AVEENO® Dermexa Fast & Long-Lasting Balm twice daily to whole body for 8 weeks vs standard routine skin-care advice. |
| Outcomes | 14 | Pre-specified primary and secondary outcomes, including the specific measurement variable (e.g., systolic blood pressure), analysis metric (e.g., change from baseline, final value, time to event), method of aggregation (e.g., median, proportion), and time point for each outcome | Methods, p. 5 primary: point prevalence of AD at 5 years; secondary: AD severity measured by SCORAD |
| Harms | 15 | How harms were defined and assessed (e.g., systematically, non-systematically) | Original intervention period previously reported; no intervention was administered during the five-year follow-up |
| Sample size | 16a | How sample size was determined, including all assumptions supporting the sample size calculation | Methods, p. 4 — original target n=242 (121/group), 80% power to detect reduction in cumulative AD from 30% to 15%; recruitment continued to n=321 for anticipated attrition. |
|  | 16b | Explanation of any interim analyses and stopping guidelines | n/a |
| Randomisation: |  |  |  |
| Sequence generation | 17a | Who generated the random allocation sequence and the method used | Methods, p. 3; original trial |
|  | 17b | Type of randomisation and details of any restriction (e.g., stratification, blocking and block size) | Methods, p. 3 — block randomisation with varying block sizes of 2, 4 and 6. |
| Allocation concealment mechanism | 18 | Mechanism used to implement the random allocation sequence (e.g., central computer/telephone; sequentially numbered, opaque, sealed containers), describing any steps to conceal the sequence until interventions were assigned | Methods, p. 3 — sequentially numbered opaque sealed envelopes; allocation concealed by study project manager. |
| Implementation | 19 | Whether the personnel who enrolled and those who assigned participants to the interventions had access to the random allocation sequence | Methods, p. 3; original trial procedures — study project manager had no patient contact or access to participant data. |
| Blinding | 20a | Who was blinded after assignment to interventions (e.g., participants, care providers, outcome assessors, data analysts) | Methods, pp. 3–4 — five-year outcome assessors were blinded to original allocation. |
|  | 20b | If blinded, how blinding was achieved and description of the similarity of interventions | Methods, p. 4 — parents instructed not to reveal original allocation during contact with blinded clinician. |
| Statistical methods | 21a | Statistical methods used to compare groups for primary and secondary outcomes, including harms | Methods, pp. 5–6 — Welch t-test, Fisher exact test, Pearson RR with 95% CI, logistic regression and MICE. |
|  | 21b | Definition of who is included in each analysis (e.g., all randomised participants), and in which group | Methods, p. 5 — analysed according to original allocation; primary complete-case population n=177 |
|  | 21c | How missing data were handled in the analysis | Methods, pp. 5–6 MICE in original n=321 cohort, 50 datasets; pooled using Rubin's rules |
|  | 21d | Methods for any additional analyses (e.g., subgroup and sensitivity analyses), distinguishing prespecified from post-hoc | Methods, pp. 5–6 adjusted complete-case, attendance/attrition analyses, MICE and exploratory longitudinal phenotypes. |
| **Results** | | |  |
| Participant flow, including flow diagram | 22a | For each group, the numbers of participants who were randomly assigned, received intended intervention, and were analysed for the primary outcome | Results/Figure 1 — 321 randomised; final five-year primary analysis n=177 (81 intervention, 96 control). |
|  | 22b | For each group, losses and exclusions after randomisation, together with reasons | Fig 1 |
| Recruitment | 23a | Dates defining the periods of recruitment and follow-up for outcomes of benefits and harms | Methods, pp. 3–4 — original recruitment April 2019–November 2020; five-year follow-up described in Methods |
|  | 23b | If relevant, why the trial ended or was stopped | N/A |
| Intervention and comparator delivery | 24a | Intervention and comparator as they were actually administered (e.g., where appropriate, who delivered the intervention/comparator, how participants adhered, whether they were delivered as intended [fidelity]) | Original trial previously reported; Methods pp. 3–4 summarises assigned intervention. |
|  | 24b | Concomitant care received during the trial for each group | Methods, p. 4 — no further intervention, specific skin-care guidance or restrictions on emollient use after 8 weeks. |
| Baseline data | 25 | A table showing baseline demographic and clinical characteristics for each group | See table 1 and Supp Table 1 |
| Numbers analysed,  outcomes and estimation | 26 | For each primary and secondary outcome, by group:   - the number of participants included in the analysis - the number of participants with available data at the outcome time point - result for each group, and the estimated effect size and its precision (such as 95% confidence interval) - for binary outcomes, presentation of both absolute and relative effect size | Results, pp. 6–7; Figures 2–4 — AD 10/81 vs 23/96; RR 0.52 (95% CI 0.26–1.02); SCORAD and sensitivity analyses also reported. |
| Harms | 27 | All harms or unintended events in each group | No harms reported |
| Ancillary analyses | 28 | Any other analyses performed, including subgroup and sensitivity analyses, distinguishing pre-specified from post-hoc | Results, p. 7; Supplementary Tables 2–3; Figure 4 — adjusted complete-case, MI and exploratory longitudinal phenotype analyses |
| **Discussion** | | |  |
| Interpretation | 29 | Interpretation consistent with results, balancing benefits and harms, and considering other relevant evidence | Discussion, pp. 7–10 interpretation in context of attrition and previous trials. |
| Limitations | 30 | Trial limitations, addressing sources of potential bias, imprecision, generalisability, and, if relevant, multiplicity of analyses | Discussion, Strengths and Limitations — attrition/selection bias, single-centre high-risk population, incomplete later emollient/topical steroid data, small AD severity sample, and exploratory non-prespecified five-year analyses |

*We strongly recommend reading this statement in conjunction with the CONSORT 2025 Explanation and Elaboration and/or the CONSORT 2025 Expanded Checklist for important clarifications on all the items. We also recommend reading relevant CONSORT extensions. See [www.consort-spirit.org](http://www.consort-spirit.org).

Citation: Hopewell S, Chan AW, Collins GS, Hróbjartsson A, Moher D, Schulz KF, et al. CONSORT 2025 Statement: updated guideline for reporting randomised trials. BMJ. 2025; 388:e081123. <https://dx.doi.org/10.1136/bmj-2024-081123>.

© 2025 Hopewell et al. This is an Open Access article distributed under the terms of the Creative Commons Attribution License (<https://creativecommons.org/licenses/by/4.0/>), which permits unrestricted use, distribution, and reproduction in any medium, provided the original work is properly cited.
